
JOURNAL INFORMATION
==============================
NLM Title Abbreviation: J Can Health Libr Assoc
Journal ID: J Can Health Libr Assoc
NLM Journal ID: 101229936
Journal ID: JCHLA

The Journal of the Canadian Health Libraries Association

EISSN: 1708-6892
Publisher: Canadian Health Libraries Association / Association des bibliotèques de la santé du Canada

ARTICLE INFORMATION
==============================
PMCID: PMC11485157
DOI: 10.29173/jchla29791
Article ID: JCHLA-45-114
Article version: 1
Subjects: Chla 2024 Conference Contributed Papers / Absc Congrès 2024 Communications Libres

CHLA 2024 CONFERENCE CONTRIBUTED PAPERS / ABSC CONGRÈS 2024 COMMUNICATIONS LIBRES

Electronic publication date: 2024 Aug 1
Publication date: 2024 Aug
Volume: 45
Issue: 2
First page: 114
Last page: 122
License: This article is distributed under a Creative Commons Attribution License: https://creativecommons.org/licenses/by/4.0/
License URL: https://creativecommons.org/licenses/by/4.0/

==============================

## CP1. Barriers and facilitators facing early career researchers and librarians in health professions when conducting systematic and scoping reviews: a mixed methods study

Lindsay Sikora
University of Ottawa

**Introduction:** This study delves into the challenges faced by early career researchers (ECRs) and librarians in health professions conducting systematic and scoping reviews. Given the pivotal role of these methodologies in evidence synthesis, understanding the unique experiences of this demographic is crucial. **Methods:** A mixed methods approach combines quantitative surveys and semi-structured interviews. The structured survey, administered to a diverse sample, examined methodological expertise, resource access, and time constraints. Simultaneously, semi-structured interviews with a subset of participants provided qualitative depth, exploring personal experiences and uncovering facilitators such as mentorship programs, collaborative networks, and specialized training. **Results:** Preliminary survey findings revealed common challenges, including limited methodological proficiency and resource constraints. Qualitative interviews contextualized these challenges, offering insights into coping strategies and nuanced facilitators that contribute to successful reviews. **Conclusion:** This research provides actionable recommendations for academic institutions, mentors, and organizations to support ECRs and librarians. By addressing identified barriers and leveraging facilitators, stakeholders can cultivate an environment conducive to high-quality evidence synthesis, advancing research and evidence-based practice in health professions. The integrated findings from both quantitative and qualitative methods offer a comprehensive understanding of the multi-faceted landscape surrounding systematic and scoping reviews in this context-based practice in health professions.

## CP2. Beyond the search: librarian-led project management of evidence syntheses

**Introduction:** Librarians traditionally support evidence synthesis (ES) by refining research questions, designing and executing searches, and providing methodological guidance. However, given the opportunity, librarians can be members of the research team beyond the search stage, and even provide leadership. Two librarians recently provided project management support for rapid living evidence syntheses for the Public Health Agency of Canada (PHAC). **Description:** COVID-END, a pan-Canadian COVID-19 evidence network, was commissioned by PHAC to produce eight rapid living evidence syntheses (LESs) on public health and social measures for reducing transmission of COVID-19. Completed by teams across Canada, each LES had three updates due at 6-week intervals. We each supported the LES on the effectiveness of masks and on the unintended consequences/outcomes of masking, respectively. Beyond the search stage, we created citation screening support documents, resolved screening conflicts, designed data extraction forms, led data extraction and verification, managed data, drafted reports, and kept each project running on time. **Outcomes:** Our teams each submitted our deliverables on time with appropriate methodological rigour. Lessons learned include the importance of negotiating scope with contractors and funders, handling tight timelines, and tips and tricks for making the most of review software and complementary programs such as Microsoft Excel. **Discussion:** By integrating librarians as project managers, our teams benefitted from the organizational skills and ES expertise many of us possess. In turn, we enhanced our project management and ES skills, and we now provide improved support to other ES teams.

### CP3. Charting equity in scholarship: strategies for libraries in addressing citation justice

Heather Cunningham
University of Toronto

**Introduction:** Citation justice, an emergent discourse in academia, addresses the systematic under-citation of women and people of colour across disciplines including the health sciences. This citation gap, reflecting structural biases rather than quality of work, has cumulative implications for career progression, grant acquisition, and decision-maker diversity in academia and healthcare. This presentation aims to delve into citation justice, review emerging efforts to reform citation practices, and suggest library strategies to mitigate these scholarly communication inequities. **Description:** Citation practices significantly influence scholarly discourse, research, and education. Currently no robust framework exists for evaluating citation biases regarding author demographics. While computational analyses attempt to assess author diversity, they have limitations. There is a growing movement among publishers to address these biases, including Citation Diversity Statements, and surveys on author race, ethnicity, and gender identity. Grass root efforts like databases of underrepresented researchers and campaigns such as "Cite Black Women" highlight the increasing commitment to address citation injustice. **Outcomes**: Libraries have a proactive role in promoting citation justice through workshops, consultations, and research guides that incorporate discussions on citation biases and diversity. Addressing or reducing bias is essential in medical literature research. Integrating citation justice when teaching comprehensive searching, research impact, and publishing can lead to fostering a more inclusive scholarly environment. **Discussion:** Addressing citation inequities requires a comprehensive approach. Libraries are key in advocating gender and racial balance in citations. By understanding the issues and sharing resources and strategies, libraries can champion citation justice, actively contributing to a more equitable scholarly communication framework.

### CP4. Data-sharing practices in publications funded by the Canadian Institutes of Health Research: implications for health sciences librarians

David Scott
University of Lethbridge

**Objective:** Data sharing in the health sciences is a known challenge as human subjects research prohibits the public sharing of personal identifying information without consent. Because of its private nature, it has been reported in the literature that health research data is difficult to find and access for reuse purposes. To better understand the current state of data sharing in a Canadian health sciences context, this study examines Canadian Institutes of Health Research (CIHR)-funded publications that provide evidence of data sharing to identify if, where, and how researchers make their data available. This study is a 3.5-year update to a previously published 2021 project. **Methods:** Using PubMed Central, we identified 8,356 articles that were published between January 1, 2020 and June 30, 2023, were funded by CIHR, and had associated data. We analyzed a sample of 368 publications (95% confidence interval) to determine if and where data were shared and what documentation was provided to facilitate data reuse. **Results:** Preliminary results indicate that 34% of articles made at least some data readily accessible, 22% indicated that data were available upon request or by application, and 44% made no research data available. Overall, 69% of sampled publications included a data availability statement, and only 9% of articles supplied documentation that would support reuse of the data. **Conclusions:** This study highlights gaps with respect to how Canadian health researchers share and document their data. This presentation will discuss these gaps and outline how health sciences librarians can support researchers in this area.

## CP5. Dealing with disruption: how generalist academic libraries are grappling with growing demands for evidence synthesis support

**Introduction:** The explosive growth of evidence synthesis projects has prompted many institutions to develop or refine their services and staffing models. This presents an additional challenge for institutions without medical schools which often lack trained librarians and resources dedicated to this service and which are seeing increased demand from many disciplines outside health sciences. This presentation will highlight approaches used by two medium-sized universities - one with liaison librarians, another with functional teams - to assess needs and develop sustainable models for supporting evidence synthesis research across diverse non-clinical disciplines. As well, the presenters will share preliminary results from a survey documenting experiences and service models at other generalist institutions. **Methods:** Following research ethics clearance, a short survey will be distributed on relevant listservs (e.g. KSIG, CAPAL) to collect information from non-health-sciences libraries about their models for supporting evidence synthesis research. **Results:** The presentation will report on expected outcomes including insights into how libraries are building structures and processes to support the growing demand for evidence synthesis research outside the traditional base of clinical health sciences. **Discussion:** Sharing the experiences of a diverse range of institutions will provide us all with insights and further questions to consider as we work to develop effective, sustainable and equitable evidence synthesis supports and services.

## CP6. Dependable during disruptions: ensuring remote access to library resources, redeploying staff, and offering relevant services during the COVID-19 pandemic

The COVID-19 pandemic impacted access to health library services, spaces, staff and resources. We conducted a bilingual Canada-wide survey in early 2023 to document the experiences of library technicians, librarians, managers, and other health library staff. The objectives of the survey were to document: (1) services provided by health library staff; (2) staffing levels in health libraries; (3) redeployment of library staff to alternate roles or activities; (4) frequently used skills or competencies; (5) challenges faced and (6) lessons learned during the pandemic. Over 280 respondents provided insights on the role of library staff during the pandemic. During the pandemic, library staff provided services such as environmental scans, literature searches, instruction sessions, and alerts to information on COVID-19 and SARS-CoV-2 so that hospital staff, public health staff or researchers could implement the most up to date evidence. Meanwhile, staffing levels at health libraries were variable between 2019-2022. In addition, survey data shows that 57% of respondents were redeployed to alternate roles or tasks and 22% of staff who were redeployed continued to perform library work simultaneously. Skills and competencies such as literature searching, project management, networking, or communication were used daily or weekly by over 40% of respondents. Many library staff identified challenges such as reduced or increased service requests, online service delivery, deployment to alternate roles and health concerns. Overall, health libraries ensured remote access to resources, redeployed staff to meet organizational priorities, and provided relevant services during the pandemic. Future considerations to improve service delivery are explored.

## CP7. Evidence-based activism: the librarian's role in grassroots challenges to healthcare policy and practice

David Gerstle
University of Toronto

**Introduction:** Evidence-Based Activism (EBA) is the agentive self-determination by patients to interrupt professional discourses on disease, disability, and therapy. In short, it is self-educated patients working to challenge healthcare policy and medical practice. Such commitments were initially identified in the public demands of 1980/90s AIDS activists (e.g. ACT UP), confronting randomized control trials in the face of the epidemic. Since then, researchers have noted (and criticized) efforts by 'laypersons' to question policy and therapy in ways typically reserved for professionals. As the name EBA implies, these movements are defined by participants' efforts to ground their cause in empirical evidence from the health sciences. Politics merges with scholarship, troubling assumptions of expertise; authority; and ownership and creation of knowledge. **Methods:** Comprehensively reviewing the EBA literature, I draw out themes elucidating how librarians can support collective action, though this may challenge traditions of professional librarianship. **Results:** As champions of evidence-based practice in the health sciences, librarians play a powerful but complicated role supporting causes to transform healthcare. We can engage underserved or silenced communities with our professional toolkits, and bring the needs of activism to discourses on information science. Yet, EBA also throws light on our limitations: our assumed neutrality, and our privileged positions as custodians of information and experts in finding it. **Discussion:** Outlining challenges and opportunities, I discuss my own experiences supporting a community-driven health network in Mississauga, Ontario focused on chronic illness, using grassroots efforts toward pop-up screening, health education, and patient empowerment.

## CP8. Green libraries: cultivating well-being and food security in academic libraries

Heather Cunningham, Glyneva Bradley-Ridout, Mikaela Gray, Catherine Duff, Margaret Nevison, Emily Woehrle
University of Toronto

**Introduction:** Integrating greenery in libraries offers benefits such as stress reduction, air quality improvement, and fostering relaxation. Numerous articles about library programming include workshops on gardening skills. Furthermore, library gardens and seed libraries can also be an innovative approach to mitigating food security. **Description:** In Spring 2022, the Gerstein Science Information Centre, University of Toronto, introduced an indoor garden, a seed library, curated monograph collection, and lending services for gardening tools. The herbs and lettuce grown in the indoor units are harvested and quickly claimed by patrons. This project aimed to enhance the well-being of patrons and staff, foster community engagement and partnerships, and align with the library's strategic plan of enhancing physical spaces and promoting health and wellness. **Outcomes:** A survey on usage and motivations for using the garden and seed library revealed significant insights into the student population. Most respondents showed a preference for edible plants over ornamental ones in the garden units. Similarly, there was a notable interest in acquiring seeds for growing food. Over 2000 seed packets were distributed to students within a year of the project launch. The responses and comments also indicated the calming effect of gardening as a hobby and for wellbeing. **Discussion:** Libraries have the potential to broaden their impact through gardens, not only as a means of psychological uplift but also as a tool to combat food insecurity.

This can be achieved through direct provision of greens and herbs, seed distribution, or educational programming in partnership with campus organizations focused on sustainable food cultivation.

### CP9. How one untamed hospital librarian seized the marketing day and disrupted the library's tempo in order to promote its value

Iveta Lewis
Holland Bloorview Hospital

**Introduction:** While fundamental in other disciplines, marketing skills appear unfamiliar to hospital librarians for many reasons. The purpose of this project is to demonstrate that a hospital library does not need massive budgets to promote services. I will show that hospital librarians can painlessly market their services to create partnerships and uphold their values. **Description:** When I started at the Holland Bloorview Kids Rehabilitation hospital library in the thick of 2020, I realized I had an unsurmountable task ahead to unveil the invisibility of the library. I will share tips on marketing a hospital library through the journey from nebulous novice marketer to eventually building a modest marketing plan. **Discussion:** Working from home during the pandemic hiatus, the library's spotlight had dimmed but there was actually a plethora of opportunities to take advantage of. I will illustrate how the pandemic ignited the promotion engine where library projects were bouncing off each other; from providing a library space as a vaccination clinic to collaborating with labs, to creating events for the IDEAA program, and to designing an exhibition for the hospital's anniversary. **Outcomes:** From the library being in a fog in 2020 to now, our library's services profile has soared exponentially. Not only does the library continue to maintain its traditional essential services, but it has also introduced many innovative ones, such as the lending devices from our research labs. It is evident that promoting library services is doable even without formal training, and achieved instead with indispensable passion to seize the day.

### CP10. Information literacy research during the COVID-19 pandemic: a systematic literature review and bibliometric analysis

Efren Torres Jr, Christine Abrigo, Zipporah Dery-Cruz
De La Salle University Manila

**Purpose:** The study aims to examine the literature on information Literacy (IL) published during the COVID-19 pandemic over the last four years, focusing on research productivity and its research impact. It also highlights the major themes and emerging trends in IL research.Specifically, it shall answer the following questions:

Q1: What is the state of research on information literacy in terms of research productivity (i.e. leading countries, authors, and sources) and research impact (i.e., citation counts) during the COVID-19 pandemic?
Q2: What structure can be derived in terms of networks of co-citation, keywords co-occurrence, and co-authorship?
Q3: What are the prevailing themes about information literacy that are being researched during the pandemic?

**Design, methodology, and approach:** Systematic literature review and bibliometric analysis were employed. Scopus database was used as the primary source of bibliographic data. **Findings:** Anticipated findings point towards the results of the synthesis of relevant literature review on information literacy research and map out research and future directions. **Originality/Value:** This study is an attempt to add to the body of bibliometric studies on information literacy intended for instructors and librarians to understand IL in depth.

### CP11. It's broken, and we gotta fix it (part 2): implementing a new library service model in a multi-site hospital network

Zack Osborne
Unity Health Toronto

**Introduction:** It can be difficult for hospital libraries to meet increasing service demands, ensuring staff time is occupied offering the services which best serve the organization, and while supporting competing priorities equitably and consistently. It's crucial for hospital libraries to adapt to the changing needs of their organizations, and to demonstrate they can align their service offerings with the strategic objectives of the organization. **Description**: This paper outlines changes made to the scope and limitations of services and activities offered by the library team at Unity Health Toronto, a multi-site academic hospital network. These changes include the introduction of a priority matrix to transparently communicate priority or precedence of service requests, the re-imagination of roles on the library team to focus service offerings on specific under-supported program areas, and the implementation of a fee-for-service model for library "specialized services". From concentrating service delivery on meaningful and intentional uses of library staff time, these changes aim to better support the education, research, and evidence-based decision making for physicians, staff and learners in an academic health network. **Outcomes:** High-level outcomes, reflections, and evaluations of success and impact will be shared at the time the paper is presented. It is hoped other hospital libraries considering modifications to how they support their communities will benefit from the approaches and strategies employed in this experience. **Discussion:** This builds on a session delivered at CHLA 2023 in which the project's methods and approaches were discussed.

### CP12. Narrative medicine in pre-medical undergraduate education: humanizing health experiences

Stephanie Sanger, Shayan Novin
McMaster University

**Introduction:** Narrative medicine (NM) is a humanistic approach to the health sciences that values individuals' stories and lived experiences. This framework has been shown to improve various competencies, including communication, perspective-taking and ethical inquiry. Graphic medicine (GM) is a subset of NM that places an emphasis on the visual expression of stories. **Description:** GM is one of the topics explored by McMaster University health sciences students in a course known as 'Praxis Pathways' In a month-long module, students read, reflect on, and present a GM story that they find personally relevant. After a group reflection, students are given the opportunity to create a short GM piece that shares their own health story. **Outcomes:** This presentation is centred in the experiences of one recent health sciences graduate who found value in NM. After completing the GM module, this student used GM to share his story growing up as a gay child. He curated his story with the intention to disrupt and bring nuance to reductive and stereotypical narratives around gay youth. **Discussion:** His learning experiences and subsequent NM-based initiatives offer insight into the successes and areas of improvement for the GM module offered in Praxis Pathways. This exploration of a student' lived experience may also shed light on the broader pedagogical considerations for NM-oriented programs delivered to undergraduate students.

## CP13. Navigating the digital frontier: unpacking strategies and assemblages in academic librarians' online teaching practices for evidence synthesis methods

Robin Parker
Dalhousie University

**Introduction:** Academic health librarians regularly teach students evidence synthesis (ES) search skills through research consultations and group sessions. Increasing demand to support student projects can challenge boundaries regarding librarians' role in ES methods and provoke decisions about how and what to teach. We know little about librarian teaching practices during remote instruction of ES methods so I aimed to untangle the organizational, technological, pedagogical, and methodological aspects of online teaching practices regarding ES methods and make visible the diverse strategies for engaging learners. **Methods:** In this digital ethnography using sociomaterial theories, 11 Canadian health librarians participated in two focus groups, eight observations of online research consultations, and five interviews. Analysis focused on tracing actions and disruptions to build relational understandings of the human, non-human, material, and immaterial entanglements in online teaching practices. **Results:** Digital learning objects, such as video tutorials and library guides, along with ES methods guidance and review-related technologies mediated librarians' online teaching practices. In response to methodological expectations and learners' demonstrated abilities, librarians calibrated their teaching to balance technical and conceptual learning objectives related to the interconnected steps of ES methods. Librarians teach searching, question formulation, and more using the affordances of various technologies, both to deliver the training and in conducting steps of the review. **Discussion:** I propose a model to frame decisions about how technologies, techniques, methods, content, and format influences online ES methods instruction, depending on the audience, setting, and context. This framework can help make explicit the often invisible labour of teaching ES methods.

## CP14. Revolutionizing learning: emerging tech spaces in the Sperber Health Sciences Library

Janice Kung, Lisa Tjosvold, Liz Dennett
University of Alberta

**Introduction:** The new Sperber Health Sciences Library at the University of Alberta opened in September 2023 and with it came the opportunity to design innovative spaces and new services. Featuring a Maker Lab (3D printing), Virtual Reality Lab, and Media Lab, these new spaces are equipped to meet the evolving educational and research needs of students and faculty. **Description:** After months of planning, the new technology services have launched and the initial enthusiasm shows that students and faculty are receptive to these technologies being housed in the library. This presentation will discuss the marketing strategies applied to promote the services, early successes, and the challenges. **Outcomes:** Preliminary analytics will be shared including usage statistics and user feedback. **Discussion:** Since the three technology services are still in their infancy, there is much room to grow and expand the programming. The team will share next steps and lessons learned from running the services for less than one year.

## CP15. Scaffolded information literacy and student perceived confidence levels

Stephanie Sanger, Denise Smith
McMaster University

When considering undergraduate general health sciences education, there is insufficient evidence that speaks to the benefits and challenges of a scaffolded information literacy curriculum. Despite some argument that the impact on students is the same regardless of how information literacy training is delivered, the call for a curriculum-integrated scaffolded training program is on the rise. This study aims to develop an understanding of the students' perceived confidence levels and skill levels when engaging with health information after four-years of formal information literacy instruction. The researchers used a mixed-methods survey to learn how students' confidence in their information literacy skills has been impacted by this curriculum. Quantitative survey questions were analyzed using a combination of statistical methods in SPSS software. Qualitative survey questions employed in vivo open coding to identify patterns and categorize themes that emerged in participant responses to open-ended questions. The survey had a response rate of 25%. 79% of survey participants recalled feeling "unconfident" or "not so confident" about how to find information when they were in their first year of study. By their final year, all participants indicated they felt "somewhat confident," (53%) or "very confident"(47%). Additional findings from the survey and qualitative findings will be discussed during the presentation. A mandatory curriculum-integrated information literacy program in undergraduate health sciences has been shown to have had a positive impact on students' confidence in searching and finding information. This study provides evidence of the benefits of integrating information literacy instruction into curricula for undergraduate students.

## CP16. Support and professional development needs for knowledge syntheses in Canadian research libraries: results from a cross-sectional survey

**Introduction:** Requests for support regarding knowledge syntheses (KS) have steadily increased and expanded beyond health disciplines. The objective of this CARL quality improvement project was to support professional development (PD) planning through understanding the needs of Canadian library workers interested in KS, regardless of discipline. **Methods:** This cross-sectional study used online bilingual surveys open to all Canadian library workers to collect baseline data about KS support and PD needs. Twenty questions were organized into four sections: 1) role at library and with KS support; 2) levels and types of KS services; 3) PD training needs and preferences; and 4) additional desired resources and supports. **Results:** 137 library workers responded (101 English and 36 French). Respondents had liaison and KS responsibilities with all listed disciplines, with approximately half working in health. Most respondents provide consultant support and mainly cover aspects related to searching and managing citations, but many also collaborate on and teach KS and cover other steps of the review process. Respondents preferred opportunities to learn about KS through structured PD, although the majority had previously built knowledge through self-directed readings and webinars. Respondents desired more time and human resources and PD on review methodologies and automation tools. **Discussion:** KS support in Canadian academic libraries is varied and expanding beyond health. Health librarians report supporting multiple disciplines, suggesting cross-over that can help build capacity into other areas through workshops and other PD. The results of this survey provide insights to guide development of KS support and capacity within and beyond health libraries.

## CP17. Undergraduate involvement in published evidence syntheses: a preliminary exploration

Fiona Inglis
Wilfrid Laurier University

**Introduction:** We are seeing an increase in undergraduate health sciences students being advised by their supervisors to publish evidence synthesis papers to enhance their medical school applications. This presents many challenges for both the students and the health sciences librarian. As we explored how best to support these students, we started by asking how often students are listed as authors in published review articles. Primary research questions include: How many review articles have Laurier affiliated authors published? How many included student authors? How do evidence syntheses vary by department? **Methods:** Six databases covering multiple disciplines were searched for titles including the word review and our institutional affiliation (n=909). Being a smaller institution, this broad scope was possible and was designed to enable analysis of disciplinary differences. Covidence was used to remove duplicates (n= 478) and support the 2-step screening process. Data extraction (n= 147) included bibliographic information, department, student status, librarian involvement, type of review, type of publication, and methodology. **Results**: Data analysis is ongoing at the time of submission, but there are some preliminary results. The most prolific departments were psychology (18%), business (17%), social work (15%) and health sciences (13%). Students contributed to 35% of the articles. The majority were doctoral students (55%) but 23% were undergraduates, mainly health sciences. Students most often wrote scoping reviews (31%). Librarians were acknowledged in 28% of the publications. **Discussion:** Understanding undergraduate involvement in evidence syntheses will enable us to develop appropriate policies and resources for sustainable and ethical support.

## CP18. Using meta-ethnography to disrupt: under-represented students' stories of (dis)embodiment and impossible professionalism in medical school

Robin Parker, Paul Cameron, Sarah Burm, Anna MacLeod, Jordin Fletcher, Olga Kits, Victoria Luong
Dalhousie University

**Introduction:** Increasing recruitment of students who are Underrepresented in Medicine (UiM) aims to align representation in medical schools to the broader population, but retention and attainment still lag. We aimed to synthesize the literature to honour the stories of UiM medical students, building a deeper understanding of their complex experiences. **Methods:** We followed the seven-step process for meta-ethnography, a critical qualitative synthesis that emphasizes richness and depth over comprehensiveness. We developed MEDLINE and Scopus searches and conducted a synthesis through reflexive team and individual processes, cultivating rich data from included studies. Involvement of a librarian, UiM students and graduates, and PhD scientists with diverse lived expertise brought rich insight into the meta-ethnographic process. **Results:** We included 37 studies describing the experiences of UiM students: racial/ethnic minorities, those with disabilities, first in family, Indigenous, LGBTQ+, and multiple groups. Synthesis generated four cross-cutting themes: "Working ten times as hard:" highlighting UiM students' additional labour; "Let me control my tone:" addressing the disciplining of UiM bodies; "I stuck out like a sore thumb:" involving othering, isolation, and visibility; "Sticking together:" exploring coping strategies. **Discussion:** Three overarching barriers complicated the stories in the literature: 1. The Disembodied doctor; 2. Impossible professionalism, which creates additional labour for underrepresented medical students in the form of: 3. Hidden work: compensation, masking, and passing. The stories from UiM students disrupt the narrative of a normative, idealized physician identity. Working on this project permitted the librarian to engage with novel methods and a diverse, interdisciplinary team.

JOURNAL INFORMATION
==============================
NLM Title Abbreviation: J Can Health Libr Assoc
Journal ID: J Can Health Libr Assoc
NLM Journal ID: 101229936
Journal ID: JCHLA

The Journal of the Canadian Health Libraries Association

EISSN: 1708-6892
Publisher: Canadian Health Libraries Association / Association des bibliotèques de la santé du Canada

ARTICLE INFORMATION
==============================
Subjects: CP = Contributed Paper

CP1. Barriers and facilitators facing early career researchers and librarians in health professions when conducting systematic and scoping reviews: a mixed methods study

Sikora Lindsay
University of Ottawa

JOURNAL INFORMATION
==============================
NLM Title Abbreviation: J Can Health Libr Assoc
Journal ID: J Can Health Libr Assoc
NLM Journal ID: 101229936
Journal ID: JCHLA

The Journal of the Canadian Health Libraries Association

EISSN: 1708-6892
Publisher: Canadian Health Libraries Association / Association des bibliotèques de la santé du Canada

ARTICLE INFORMATION
==============================
Subjects: CP = Contributed Paper

CP2. Beyond the search: librarian-led project management of evidence syntheses

Boulos Leah 1
Langman Erin 2
1 Dalhousie University
2 University of Saskatchewan

JOURNAL INFORMATION
==============================
NLM Title Abbreviation: J Can Health Libr Assoc
Journal ID: J Can Health Libr Assoc
NLM Journal ID: 101229936
Journal ID: JCHLA

The Journal of the Canadian Health Libraries Association

EISSN: 1708-6892
Publisher: Canadian Health Libraries Association / Association des bibliotèques de la santé du Canada

ARTICLE INFORMATION
==============================
Subjects: CP = Contributed Paper

CP3. Charting equity in scholarship: strategies for libraries in addressing citation justice

Cunningham Heather
University of Toronto

JOURNAL INFORMATION
==============================
NLM Title Abbreviation: J Can Health Libr Assoc
Journal ID: J Can Health Libr Assoc
NLM Journal ID: 101229936
Journal ID: JCHLA

The Journal of the Canadian Health Libraries Association

EISSN: 1708-6892
Publisher: Canadian Health Libraries Association / Association des bibliotèques de la santé du Canada

ARTICLE INFORMATION
==============================
Subjects: CP = Contributed Paper

CP4. Data-sharing practices in publications funded by the Canadian Institutes of Health Research: implications for health sciences librarians

Scott David
University of Lethbridge

JOURNAL INFORMATION
==============================
NLM Title Abbreviation: J Can Health Libr Assoc
Journal ID: J Can Health Libr Assoc
NLM Journal ID: 101229936
Journal ID: JCHLA

The Journal of the Canadian Health Libraries Association

EISSN: 1708-6892
Publisher: Canadian Health Libraries Association / Association des bibliotèques de la santé du Canada

ARTICLE INFORMATION
==============================
Subjects: CP = Contributed Paper

CP5. Dealing with disruption: how generalist academic libraries are grappling with growing demands for evidence synthesis support

Inglis Fiona 1
Yates Elizabeth 2
1 Wilfrid Laurier University;
2 Brock University

JOURNAL INFORMATION
==============================
NLM Title Abbreviation: J Can Health Libr Assoc
Journal ID: J Can Health Libr Assoc
NLM Journal ID: 101229936
Journal ID: JCHLA

The Journal of the Canadian Health Libraries Association

EISSN: 1708-6892
Publisher: Canadian Health Libraries Association / Association des bibliotèques de la santé du Canada

ARTICLE INFORMATION
==============================
Subjects: CP = Contributed Paper

CP6. Dependable during disruptions: ensuring remote access to library resources, redeploying staff, and offering relevant services during the COVID-19 pandemic

Sharma Minakshi 1
Nicol Marie-Hélène 2
Faulkner Amy 3
Charron Danielle
Harron Tanya 4
1 Toronto Public Health;
2 Université de Montréal;
3 Simcoe Muskoka District Health Unit;
4 Wellington-Dufferin-Guelph Public Health

JOURNAL INFORMATION
==============================
NLM Title Abbreviation: J Can Health Libr Assoc
Journal ID: J Can Health Libr Assoc
NLM Journal ID: 101229936
Journal ID: JCHLA

The Journal of the Canadian Health Libraries Association

EISSN: 1708-6892
Publisher: Canadian Health Libraries Association / Association des bibliotèques de la santé du Canada

ARTICLE INFORMATION
==============================
Subjects: CP = Contributed Paper

CP7. Evidence-based activism: the librarian's role in grassroots challenges to healthcare policy and practice

Gerstle David
University of Toronto

JOURNAL INFORMATION
==============================
NLM Title Abbreviation: J Can Health Libr Assoc
Journal ID: J Can Health Libr Assoc
NLM Journal ID: 101229936
Journal ID: JCHLA

The Journal of the Canadian Health Libraries Association

EISSN: 1708-6892
Publisher: Canadian Health Libraries Association / Association des bibliotèques de la santé du Canada

ARTICLE INFORMATION
==============================
Subjects: CP = Contributed Paper

CP8. Green libraries: cultivating well-being and food security in academic libraries

Cunningham Heather
Bradley-Ridout Glyneva
Gray Mikaela
Duff Catherine
Nevison Margaret
Woehrle Emily
University of Toronto

JOURNAL INFORMATION
==============================
NLM Title Abbreviation: J Can Health Libr Assoc
Journal ID: J Can Health Libr Assoc
NLM Journal ID: 101229936
Journal ID: JCHLA

The Journal of the Canadian Health Libraries Association

EISSN: 1708-6892
Publisher: Canadian Health Libraries Association / Association des bibliotèques de la santé du Canada

ARTICLE INFORMATION
==============================
Subjects: CP = Contributed Paper

CP9. How one untamed hospital librarian seized the marketing day and disrupted the library's tempo in order to promote its value

Lewis Iveta
Holland Bloorview Hospital

JOURNAL INFORMATION
==============================
NLM Title Abbreviation: J Can Health Libr Assoc
Journal ID: J Can Health Libr Assoc
NLM Journal ID: 101229936
Journal ID: JCHLA

The Journal of the Canadian Health Libraries Association

EISSN: 1708-6892
Publisher: Canadian Health Libraries Association / Association des bibliotèques de la santé du Canada

ARTICLE INFORMATION
==============================
Subjects: CP = Contributed Paper

CP10. Information literacy research during the COVID-19 pandemic: a systematic literature review and bibliometric analysis

Torres Efren Jr
Abrigo Christine
Dery-Cruz Zipporah
De La Salle University Manila

JOURNAL INFORMATION
==============================
NLM Title Abbreviation: J Can Health Libr Assoc
Journal ID: J Can Health Libr Assoc
NLM Journal ID: 101229936
Journal ID: JCHLA

The Journal of the Canadian Health Libraries Association

EISSN: 1708-6892
Publisher: Canadian Health Libraries Association / Association des bibliotèques de la santé du Canada

ARTICLE INFORMATION
==============================
Subjects: CP = Contributed Paper

CP11. It's broken, and we gotta fix it (part 2): implementing a new library service model in a multi-site hospital network

Osborne Zack
Unity Health Toronto

JOURNAL INFORMATION
==============================
NLM Title Abbreviation: J Can Health Libr Assoc
Journal ID: J Can Health Libr Assoc
NLM Journal ID: 101229936
Journal ID: JCHLA

The Journal of the Canadian Health Libraries Association

EISSN: 1708-6892
Publisher: Canadian Health Libraries Association / Association des bibliotèques de la santé du Canada

ARTICLE INFORMATION
==============================
Subjects: CP = Contributed Paper

CP12. Narrative medicine in pre-medical undergraduate education: humanizing health experiences

Sanger Stephanie
Novin Shayan
McMaster University

JOURNAL INFORMATION
==============================
NLM Title Abbreviation: J Can Health Libr Assoc
Journal ID: J Can Health Libr Assoc
NLM Journal ID: 101229936
Journal ID: JCHLA

The Journal of the Canadian Health Libraries Association

EISSN: 1708-6892
Publisher: Canadian Health Libraries Association / Association des bibliotèques de la santé du Canada

ARTICLE INFORMATION
==============================
Subjects: CP = Contributed Paper

CP13. Navigating the digital frontier: unpacking strategies and assemblages in academic librarians' online teaching practices for evidence synthesis methods

Parker Robin
Dalhousie University

JOURNAL INFORMATION
==============================
NLM Title Abbreviation: J Can Health Libr Assoc
Journal ID: J Can Health Libr Assoc
NLM Journal ID: 101229936
Journal ID: JCHLA

The Journal of the Canadian Health Libraries Association

EISSN: 1708-6892
Publisher: Canadian Health Libraries Association / Association des bibliotèques de la santé du Canada

ARTICLE INFORMATION
==============================
Subjects: CP = Contributed Paper

CP14. Revolutionizing learning: emerging tech spaces in the Sperber Health Sciences Library

Kung Janice
Tjosvold Lisa
Dennett Liz
University of Alberta

JOURNAL INFORMATION
==============================
NLM Title Abbreviation: J Can Health Libr Assoc
Journal ID: J Can Health Libr Assoc
NLM Journal ID: 101229936
Journal ID: JCHLA

The Journal of the Canadian Health Libraries Association

EISSN: 1708-6892
Publisher: Canadian Health Libraries Association / Association des bibliotèques de la santé du Canada

ARTICLE INFORMATION
==============================
Subjects: CP = Contributed Paper

CP15. Scaffolded information literacy and student perceived confidence levels

Sanger Stephanie
Smith Denise
McMaster University

JOURNAL INFORMATION
==============================
NLM Title Abbreviation: J Can Health Libr Assoc
Journal ID: J Can Health Libr Assoc
NLM Journal ID: 101229936
Journal ID: JCHLA

The Journal of the Canadian Health Libraries Association

EISSN: 1708-6892
Publisher: Canadian Health Libraries Association / Association des bibliotèques de la santé du Canada

ARTICLE INFORMATION
==============================
Subjects: CP = Contributed Paper

CP16. Support and professional development needs for knowledge syntheses in Canadian research libraries: results from a cross-sectional survey

Parker Robin 1
Clar Monique 2
Bhatnagar Neera 3
Labelle Patrick R. 4
Premji Zahra 5
1 Dalhousie University;
2 Université de Montrèal;
3 McMaster University;
4 University of Ottawa;
5 University of Victoria

JOURNAL INFORMATION
==============================
NLM Title Abbreviation: J Can Health Libr Assoc
Journal ID: J Can Health Libr Assoc
NLM Journal ID: 101229936
Journal ID: JCHLA

The Journal of the Canadian Health Libraries Association

EISSN: 1708-6892
Publisher: Canadian Health Libraries Association / Association des bibliotèques de la santé du Canada

ARTICLE INFORMATION
==============================
Subjects: CP = Contributed Paper

CP17. Undergraduate involvement in published evidence syntheses: a preliminary exploration

Inglis Fiona
Wilfrid Laurier University

JOURNAL INFORMATION
==============================
NLM Title Abbreviation: J Can Health Libr Assoc
Journal ID: J Can Health Libr Assoc
NLM Journal ID: 101229936
Journal ID: JCHLA

The Journal of the Canadian Health Libraries Association

EISSN: 1708-6892
Publisher: Canadian Health Libraries Association / Association des bibliotèques de la santé du Canada

ARTICLE INFORMATION
==============================
Subjects: CP = Contributed Paper

CP18. Using meta-ethnography to disrupt: under-represented students’ stories of (dis)embodiment and impossible professionalism in medical school

Parker Robin
Cameron Paul
Burm Sarah
MacLeod Anna
Fletcher Jordin
Kits Olga
Luong Victoria
Dalhousie University

